# Mice subjected to *aP2-Cre* mediated ablation of microsomal triglyceride transfer protein are resistant to high fat diet induced obesity

**DOI:** 10.1186/s12986-016-0061-6

**Published:** 2016-01-08

**Authors:** Ahmed Bakillah, M. Mahmood Hussain

**Affiliations:** Department of Cell Biology, SUNY Downstate Medical Center, 450 Clarkson Avenue, Brooklyn, NY 11203 USA; Department of Pediatrics, SUNY Downstate Medical Center, 450 Clarkson Avenue, Brooklyn, NY 11203 USA; VA New York Harbor Healthcare System, Brooklyn, NY 11209 USA

**Keywords:** Adipose, Lipoproteins, Lipid droplets, Inflammation, Macrophages, Obesity, Microsomal triglyceride transfer protein, High fat diet

## Abstract

**Background:**

Microsomal triglyceride transfer protein (MTP) is essential for the assembly of lipoproteins. MTP has been shown on the surface of lipid droplets of adipocytes; however its function in adipose tissue is not well defined. We hypothesized that MTP may play critical role in adipose lipid droplet formation and expansion.

**Methods:**

Plasmids mediated overexpression and siRNA mediated knockdown of *Mttp* gene were performed in 3T3-L1 pre-adipocytes to evaluate the effects of MTP on cell differentiation and triglyceride accumulation. Adipose-specific knockdown of MTP was achieved in mice bybreeding MTP floxed (*Mttp*^*fl/fl*^) mice with aP2-Cre recombinase transgenic mice. Adipose-specific MTP deficient (*A-Mttp*^*-/-*^) mice were fed 60 % high-fat diet (HFD), and the effects of MTP knockdown on body weight, body fat composition, plasma and tissues lipid composition, glucose metabolism, lipogenesis and intestinal absorption was studied. Lipids were measured in total fasting plasma and size fractionated plasma using colorimetric assays. Gene expression was investigated by Real-Time quantitative PCR. All data was assessed using t-test, ANOVA.

**Results:**

MTP expression increased during early differentiation in 3T3-L1 cells, and declined later. The increases in MTP expression preceded PPARγ expression. MTP overexpression enhanced lipid droplets formation, and knockdown attenuated cellular lipid accumulation. These studies indicated that MTP positively affects adipogenesis.

The ablation of the Mttp gene using aP2-Cre (*A-Mttp*^*-/-*^) in mice resulted in a lean phenotype when fed a HFD. These mice had reduced white adipose tissue compared with wild-type *Mttp*^*fl/fl*^ mice. The adipose tissue of *A-Mttp*^*-/-*^ mice had increased number of smaller size adipocytes and less macrophage infiltration. Further, these mice were protected from HFD-induced fatty liver. The *A-Mttp*^*-/-*^ mice had moderate increase in plasma triglyceride, but normal cholesterol, glucose and insulin levels. Gene expression analysis showed that the adipose tissue of the *A-Mttp*^*-/-*^ mice had significantly lower mRNA levels of PPARγ and its downstream targets.

**Conclusion:**

These data suggest that MTP might modulate adipogenesis by influencing PPARγ expression, and play a role in the accretion of lipids to form larger lipid droplets. Thus, agents that inactivate adipose MTP may be useful anti-obesity drugs.

## Background

The prevalence of obesity has steadily increased over the past three decades both in the United States and Worldwide, and it is associated with many diseases such as diabetes, dyslipidemia, cardiovascular disease and certain types of cancer [1–5]. Adipocytes are highly specialized cells that play an important role in the storage and utilization of fat as an energy source [6, 7]. Their main function is to synthesize and store triglycerides when there are excess calories, and mobilize this fat to other organs when caloric intake is low. Tremendous efforts with a focus on energy metabolism and lipid homeostasis have been made to obtain information about different proteins that contribute to obesity and its related complications [3, 8, 9].

Microsomal triglyceride transfer protein (MTP) assists in the assembly of triglyceride-rich lipoproteins in hepatic, intestinal, cardiac, embryonic yolk sac and glomeruli [10–13]. Besides these cells, MTP activity has been documented in adipocytes [14, 15] and macrophages [16] that do not synthesize lipoproteins. In adipose tissue, the amounts of MTP are ~ 1 % of the liver [14]. Similar low levels are also seen in macrophages suggesting that MTP expression is significantly lower in cells that do not assemble lipoproteins. In macrophages, MTP is involved in the biosynthesis of CD1 proteins that present glycolipid antigens to natural killer T (NKT) cells [12, 15, 16]. In apoB-producing cells, MTP physically interacts with apoB [10, 17, 18], whereas in macrophages it interacts with CD1d [16]. In both cases, these proteins are loaded with lipids. In case of apoB, several hundred lipid molecules are added to generate lipoproteins. By contrast, one phospholipid molecule is added to the nascent CD1 proteins in the endoplasmic reticulum (ER) [12, 19, 20]. MTP could play a similar role in adipocytes. In fact, a recent study revealed that MTP might play a role in the presentation of endogenous lipid antigens to NKT cells in adipocytes [21].

In mice, two MTP isoforms have been identified [14–16]. These two isoforms differ by two amino acids at the N-terminus and show no differences in their lipid transfer activity. MTP B is the major isoform in adipocytes [14, 15]. In enterocytes, hepatocytes and macrophages, MTP A is mainly present in the ER [16, 22, 23]. By contrast, MTP B has been suggested to be mainly present in the Golgi of non-differentiated 3T3-L1 cells [14, 15]. After differentiation, MTP was also seen on the surface of small lipid droplets [14]. Thus, there are significant differences about the subcellular localization of MTP in adipocytes as opposed to enterocytes and hepatocytes. The present study was designed to investigate potential role of MTP in adipocyte differentiation and lipid droplet formation. We hypothesized that adipose tissue MTP might participate in lipid droplet expansion by assisting in the fusion process of smaller lipid droplets and increasing their size. To test this, we used preadipocytes, 3T3-L1, and ablated the *Mttp* gene by breeding *Mttp*-floxed mice (*Mttp*^*fl/fl*^) with *aP2-Cre* mice. Our studies demonstrate, for the first time, that MTP contributes to the biogenesis of larger lipid droplets in the adipose tissue.

## Methods

### Cell culture and induction of differentiation of 3T3-L1 pre-adipocytes

Extensive information has been obtained using cell culture models such as 3T3-L1 cells to study adipocyte differentiation [8, 24]. Culture and differentiation of 3T3-L1 preadipocytes were performed as described in previous studies [25–27]. Briefly, 3T3-L1 cells, kindly provided by Dr. Brasaemle of the Rutgers University, were seeded at 2 × 10^5^/well of 6-well plates and cultured in DMEM medium supplemented with 10 % FBS, 30 μM biotin, 20 mM of glutamine, 1U/ml of penicillin/streptomycin. After confluence was reached, cell differentiation was induced by culturing in DMEM containing 1 μM insulin, 1 μM dexamethasone, and 0.5 mM of 3-isobutyl-1-methylxanthine (IBMX). After 48 h, induction media was removed and replaced with DMEM containing 10 % FBS, 1 μM insulin, 20 mM of glutamine and 1U/ml of penicillin/streptomycin, and renewed every 2 days. Lipid droplets in differentiated adipocytes (day 6 to day 10) were identified under microscope and after Oil Red O staining. In subsequent experiments, MTP inhibitors (MTPi), kindly provided by Dr. David Gordon of Bristol-Myers Squibb, were added to the media after cell induction. Fresh doses of MTPi were renewed every 2 days (D0-D10).

### Animals and diets

Transgenic mice expressing Cre recombinase under the control of rat *aP2* promoter (Tg (Fabp4-cre)1Rev or *aP2-Cre* mice) were purchased from The Jackson Laboratory. In these mice, the expression of Cre was driven by the adipocyte protein 2 (aP2)-fatty acid binding protein 4 (FABP4) promoter. The C57/BL6J mice containing the floxed MTP allele *Mttp*^*fl/fl*(exon5,6)^ [28] were bred with *aP2-Cre* mice to generate *aP2-Cre*^*+*^*/Mttp*^*fl/fl*(exon5,6)^ mice, thereafter referred to as *A-Mttp*^*−/−*^ mice. Due to the deletion of exons 5 and 6 these mice are deficient in both the A and B MTP isoforms that arise due to alternate splicing of the exon 1 [15, 16]. Age and sex matched eight-weeks old female and male wild type (WT) *Mttp*^*fl/fl*(exon5,6)^ and *A-Mttp*^*−/−*^ mice were fed a high fat diet (Rodent Diet with 60 % kcal% fat., catalog #D12492, Research Diets, Inc., New Brunswick-NJ) for 15 and 24 weeks. All studies were approved by the Institutional Animal Care and Use Committee of State University of New York Downstate Medical Center.

### Measurements of plasma lipids and transaminases (ALT/AST)

Total cholesterol and triglyceride (Thermo-Fisher Scientific), free cholesterol and free fatty acids (Wako Chemicals), and glycerol (Sigma) levels were measured in the plasma and tissues using commercial kits. Glycerol levels were subtracted from triglyceride levels. Plasma lipoproteins were separated by gel filtration (flow rate of 0.2 ml/min) using a superpose-6-column, and 200-μl fractions were collected. Fractions were used to measure cholesterol and triglycerides. To measure plasma transaminases, 2–5 μl of plasma from *Mttp*^*fl/fl*^ and *A-Mttp*^*−/−*^ mice were used for aspartate aminotransferase (AST) and alanine aminotransferase (ALT) assays using specific kits from BioTron Diagnostics (Hemet, CA) according to the manufacturer’s guidelines.

### Determination of MTP activity

Cells and small pieces (100 mg) of white adipose, liver and proximal intestine (∼1 cm) were homogenized in low salt buffer (1 mM Tris–HCl, pH 7.6, 1 mM EGTA, and 1 mM MgCl_2_) and centrifuged, and supernatants were used for protein determination and the MTP assay [29, 30] using a commercial kit (Chylos, Inc.).

### Cell transfection with plasmid DNA or siRNA

Plasmids pCMV6 (empty vector), pRcMTP expressing full length MTP, and MTP-FLAG have been previously described [30, 31]. SiRNA directed against mouse MTP (Sc-45276, MTP-siRNA) and nonspecific control siRNA (Sc-37007, Control) were obtained from Santa Cruz Biotechnology. Adipocytes were transfected in suspension as described by Kilroy et al. [32]. Briefly, 3T3-L1 cells were induced for 2 days and partially differentiated until day 4. At day 4, cells were trypsinized, resuspended in DMEM-high glucose containing 10 % FBS and gently centrifuged. Cell pellets were resuspended in 200 μl of OptiMEM (Gibco, Life Technologies) and incubated at 37 °C with equal volume of lipofectamine complexes (RNAiMax; Life Technologies) containing siRNA (100 nM) or plasmid DNA (1 μg/ml). After 20 min incubation, the mixtures were plated in 6-well plates and incubated at 37 °C in 95 % O_2_ and 5 % CO_2_ atmosphere. After 24 h-72 h post-transfection, cells were harvested to measure protein, lipid, and MTP activity.

### Tissue histology and immunohistochemistry

Samples of white adipose tissue and liver were fixed by overnight immersion in 4 % paraformaldehyde in 0.1 M phosphate buffer, dehydrated in 30 % sucrose, embedded in M1 cryo-preservation media at −20 °C, and stored at −80 °C. To assess adipose tissue and liver morphology, sections (5–7 μm) were placed on Tissue-Tack (Polysciences) slides and stained with hematoxylin and eosin (H & E) [33, 34]. To assess fat cell number, white adipose tissue was incubated with 1 mg/ml of type I collagenase (Worthington Biochemical) for 1 h at 37 °C in Krebs buffer solution containing 1 % BSA and 200 nM adenosine and 50 μg/ml glucose and filtered through cheesecloth [35, 36]. Methylene blue was added to cell suspension and an aliquot was counted under microscope using a hemocytometer. To determine adipocyte size and number, stained samples were photographed using spot digital camera interfaced to an Olympus microscope and a Macintosh computer. Images of 100 adipocytes from each fat sample were converted into a binary format with public domain National Institutes of Health Image Program (Image J Basics, version 1.38). The data were averaged per section and then per animal. Other sections were used for immunostaining of adipose tissue macrophages using specific marker F4/80 [37–39]. Briefly, sections were incubated for 2 h at 25 °C with anti-mouse F4/80 primary antibody (1:100 in 1 % BSA/PBS; Abcam #ab6640) followed by the application of HRP-conjugated rabbit anti-goat IgG secondary antibody (Thermo Scientific, Pierce antibodies #31433). Histochemical reactions were performed using the EnVision™ Doublestain System (Dako) and counterstained with Hematoxylin. Sections were examined with a light microscopy, and digital images were taken to evaluate macrophage contents.

### Oil Red O staining and measurement of cellular lipid accumulation

3T3-L1 cells were cultured and differentiated on sterile cover slips placed in 6-well plates. Differentiated cells were washed with PBS, fixed in 4 % formaldehyde solution for 15 min, washed with PBS and then stained for 1 h with Oil Red-O (Sigma-Aldrich Chemical) stain solution 0.5 % in isopropanol:water (60:40) filtered through a 0.2 μm filter. After several washes with water, pictures of samples were taken using a microscope [40]. Adipocyte differentiation was monitored by measuring cellular lipid accumulation using the same Oil Red-O staining method [40, 41]. Briefly, differentiated cells on 6-well plates were fixed with 10 % ice-cold formalin in PBS for 2 h and stained by adding Oil Red-O working solution for 10 min. Cells were thoroughly rinsed with water and the red dye retained by cells was eluted by adding isopropanol for 1 h. Aliquots were transferred to wells of a 96-well plate and optical density was measured at 500 nm wavelength. Blank samples (with no cells) were treated similarly and background values were subtracted. In subsequent experiments when the Oil Red O stain is not used, cells were washed with PBS, lysed by polytron homogenizer in low salt buffer (1 mm Tris–HCl, pH 7.6, 1 mm EGTA, and 1 mm MgCl_2_), and cellular lipids content were measured using commercial kits (Waco Chemicals; Thermo-Fisher Scientific).

### Glucose and insulin tolerance tests

Whole body plasma glucose and insulin levels were measured after overnight fasting. For glucose tolerance test (GTT), mice were fasted overnight (~16 h) with full access of water, and injected intraperitoneally with 2 mg of glucose in saline solution/g of body weight. Blood glucose levels were measured at the indicated time points before and after glucose challenge. For insulin tolerance test (ITT), mice were fasted for 4 h prior to intraperitoneal injection of insulin (Novolin R, Novo Nordisk, Denmark; 0.75 U/kg body weight). Tail vein blood glucose was collected at the indicated times using a One-Touch basic glucometer (Bayer).

### Fatty acid oxidation and de novo lipogenesis

For fatty acid oxidation measurements, 100 mg of tissues (sliced in small pieces) were incubated with [^14^C]-Oleic acid (0.3 μCi) for 2 h at 37 °C, and the radiolabeled CO_2_ was captured with filter paper soaked with phenylethylamine [42]. Radiolabel was quantified using a scintillation counter (Beckman LS 6000TA). Oxidation was presented per mg of protein. For de novo lipogenesis, 50 mg of tissue slices were placed in 1 ml Krebs-Ringer-Bicarbonate Hepes buffer containing 4 % (wt/vol) fatty acid-free BSA fraction V (Sigma), 3 mM glucose, 0.5 mM acetate and 1 μCi [1-^14^C]-Acetate (56 mCi/mmol) at 37 °C for 2 h under 95 % O_2_ – 5 % CO_2._ The reaction was terminated by the addition of 40 μl 10 N H_2_SO_4,_ and radiolabel incorporation into lipids was determined [43].

### Postprandial triglyceridemic response and intestinal triglyceride secretion

Mice were fasted for 4 h prior to the fat challenge. To study postprandial response, mice were administered with 200 μl olive oil (via oral gavage). Plasma triglyceride concentration was determined at baseline (time 0) and at 2 h and 4 h. To assess intestinal triglyceride production, fasted mice (4 h) were injected with P407 (i.p.) to block lipase activity in the circulation. After 30 min, mice were orally administered with 200 μl olive oil, and plasma triglycerides were measured before and at 2 h and 4 h post fat load challenge.

### Gene expression and qPCR

Total RNA from cells and tissues were isolated using TRIzol™ (Invitrogen). The purity of RNA was assessed by the *A*_260_/*A*_280_ ratio. RNA preparations with *A*_260_/*A*_280_ ratios more than 1.7 were used for cDNA synthesis. The first strand cDNA was synthesized using Omniscript RT (Qiagen) kit. Each reaction of quantitative PCR was carried out in a volume of 20 μl, consisting of 5 μl of cDNA sample (1:100 dilution of the first strand cDNA sample) and 15 μl of PCR master mix solution containing 1× PCR buffer (qPCR™ core kit for SYBR Green I, Eurogentec). The PCR was carried out by incubating the reaction mixture first for 10 min at 95 °C followed by 40 cycles of 15 s incubation at 95 °C and 1 min at 60 °C in an ABI 7000 SDS PCR machine. Data were analyzed using ΔΔ*C*_*T*_ method, according to the manufacturer’s instructions using the following equation: 2^[*CT* house keeping gene − *CT* target gene]^. Final results are presented as arbitrary units that were normalized to 18S mRNA.

### Statistical analyses

Data are presented as mean ± S.D. Statistical significance (*p* < 0.05) was determined using Student’s *t* test or one-way analysis of variance (GraphPad Prism 5).

## Results

### MTP activity changes during differentiation of 3T3-L1 cells do not correlate with adipogenesis

**Fig. 1:**
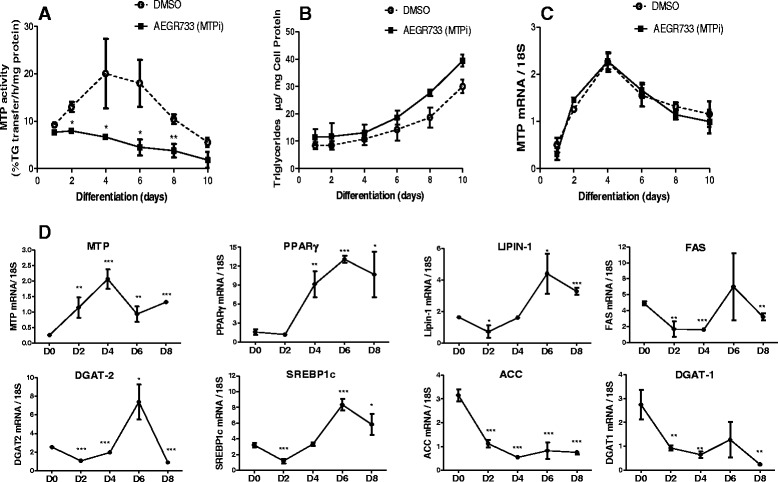
Changes in MTP activity and lipid droplets formation during differentiation of 3T3-L1 preadipocytes into mature adipocytes. Cells were first induced with INS/DEX/IBMX for 2 days and then differentiated in 10 % FBS media containing insulin for 10 days. Following induction, differentiated cells were treated either with DMSO (*dotted lines*) or in the presence of 1 μM of MTPi [Lomitapide (AEGR-733); *solid lines*] and collected every 2 days until adipocytes full maturation (*D10*). Cells were homogenized and assayed for MTP activity (**a**), triglycerides content (**b**), and Mttp gene expression (**c**). In subsequent experiment, expression profiles of different genes were analyzed on different days (**d**). Relative mRNA levels were measured by qPCR and normalized to 18S RNA. Results are mean of triplicates ± SD. Significance is represented by *, *p* < 0.05 and **, *p* < 0.01. Data are representative of two independent experiments

To understand how adipose tissue MTP might play a role in adipogenesis, we used 3T3-L1 cells that are routinely used to study adipocyte differentiation and adipogenesis. We studied changes in MTP activity during differentiation of these cells into adipocytes. Cells were treated with inducers of differentiation for 2 days and then cultured for 10 days. MTP activity increased in control cells (DMSO only) by ~ 50-100 % until day 4, stayed high till day 6, and then progressively declined until day 10 (Fig. 1a). We then measured changes in cellular lipids and droplet formation during this period. Intracellular cholesterol and fatty acids did not change significantly (data not shown). However, cellular triglyceride levels increased progressively during differentiation of control cells (Fig. 1b). Oil Red O staining also suggested higher amounts of lipids in differentiated cells (data not shown). These data suggest that MTP activity transiently increases during early differentiation and then declines later.

### Lipid transfer activity is not required for triglyceride accumulation during differentiation of 3T3 L1 cells

Next, we sought to test the hypothesis that chemical inhibition of MTP lipid transfer activity in adipocytes would interfere with cell differentiation and/or adipogenesis. To test this, we treated 3T3-L1 cells with a potent pharmacological MTPi during differentiation. Supplementation of MTPi significantly reduced MTP activity during differentiation (Fig. 1a). MTPi did not affect intracellular triglyceride accumulation (Fig. 1b). No intracellular accumulation of cholesterol or free fatty acids was observed after MTPi treatment (Data not shown). These studies indicate that inhibition of MTP lipid transfer activity does not affect triglyceride accumulation during differentiation of 3T3-L1 cells.

### MTP expression is induced early during differentiation

To understand reasons for increased MTP activity during early differentiation, we measured MTP mRNA levels (Fig. 1c). MTP mRNA levels increased by two-fold, early during differentiation, and peaked at day four. In addition, MTPi had no effect on *Mttp* gene expression. The increases in MTP mRNA preceded increases in other adipocyte genes such as PPARγ, Lipin and Fas (Fig. 1d). Thus, MTP appears to be an early response gene induced during preadipocyte differentiation.

### Knockdown and overexpression of MTP reduces and increases cellular triglyceride during differentiation of 3T3-L1 preadipocytes

**Fig. 2:**
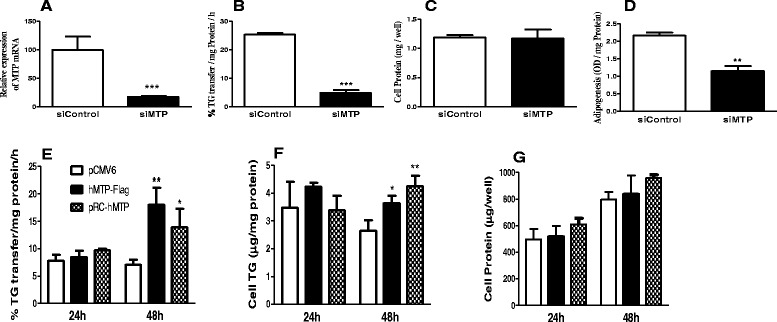
RNAi-based MTP gene silencing and MTP overexpression in differentiated 3T3-L1 cells modulates adipogenesis. Cells were partially differentiated in DMEM containing 10 % FBS until D4. On day 4, cells were transfected with scrambled siRNA or siRNA for MTP and replated. Three days postransfection (day 7), cells were collected and assessed for MTP expression (**a**), MTP activity (**b**), total cell protein content (**c**), and lipid droplet formation (**d**). In a different experiment, cells were transfected using lipofectamine reagent and 1 μg of cDNA of empty vector (*pCMV6*) or human MTP (hMTP-Flag or pRC-hMTP) for 24 h and 48 h transfection. Cells were harvested in homogenization buffer to determine MTP activity (**e**), intracellular triglycerides (**f**), and total cell protein (**g**). Results shown are representative of two independent experiments. Values are mean of triplicates ± SD. *, *p* < 0.05 and **, *p* < 0.01

To address whether early induction of MTP is important for preadipocyte differentiation, we reduced and increased MTP expression using siRNA and expression plasmids, respectively. siMTP significantly reduced MTP expression (Fig. 2a) and activity (Fig. 2b) by 82 % to 80 %, respectively, without altering total cell protein (Fig. 2c). Reductions in MTP were associated with 47 % decrease in lipid accumulation after Oil Red O staining (Fig. 2d). We also transfected differentiated 3T3-L1 cells with two different plasmids expressing human MTP cDNA resulting in significant increases in MTP activity (Fig. 2e) and cellular triglyceride after 48 h of transfection (Fig. 2f) with no effect on cellular protein levels (Fig. 2g). These studies indicated that changes in MTP levels directly correlate with changes in cellular triglyceride levels*.*

### AP2-Cre-mediated ablation of the Mttp gene in the adipose tissue results in lean mouse phenotype

**Fig. 3:**
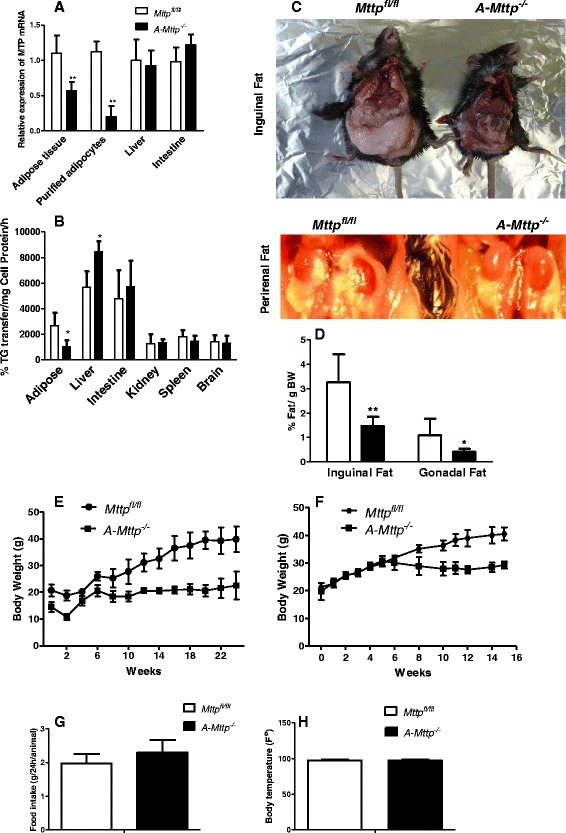
*aP2-Cre* mediated MTP ablation reduces body weight and lipid accumulation in the adipose tissue. *Mttp* ^*fl/fl*^ and *A-Mttp* ^*−/−*^ mice were fed a HFD for 24 weeks. At the end of the experiment, control and *A-Mttp* ^*−/−*^ mice were sacrificed and organs collected for MTP mRNA measurement (**a**) and MTP activity (**b**). Representative pictures of visceral fat pads and animal morphology are shown in panel (**c**). Percentages of fat in different adipose tissues by gram body weight are plotted in (**d**). Body weights were recorded every 2 weeks during the experiment using female (**e**) and male mice (**f**). Data for food consumption and body temperature are represented in (**g & h**). Values are mean of 5–7 mice ± SD from each group. *, *p* < 0.05; **, *p* < 0.01; and ***, *p* < 0.001

To study the role of MTP, we crossed *Mttp*^*fl/fl*^ mice with *aP2-Cre* mice and generated *A-Mttp*^*−/−*^ mice deficient in both the MTP isoforms. *A-Mttp*^*−/−*^ mice had 50 % to 80 % less MTP mRNA levels in the adipose tissue compared to controls (Fig. 3a). However, MTP mRNA levels were not different in the liver and intestine of WT and *A-Mttp*^*−/−*^ mice. Next, we measured MTP activity in different tissues (Fig. 3b). AP2 is known to be expressed in the brain and spleen (rich in macrophages), but MTP activity was not reduced in these tissues in *A-Mttp*^*−/−*^ mice compared to WT (Fig. 3b). However, > 50 % reduction in MTP activity was found in inguinal fat pads. Thus, *A-Mttp*^*−/−*^ mice have significant MTP deficiency in the adipose tissue.

On a chow diet, *Mttp*^*fl/fl*^ and *A-Mttp*^*−/−*^ mice gained similar weight (not shown). Next, we studied the development of obesity in *Mttp*^*fl/fl*^ and *A-Mttp*^*−/−*^ mice. *A-Mttp*^*−/−*^ mice appeared smaller in size and were leaner mainly due to significant absence of abdominal fat compared with control mice (Fig. 3c). Consistent with this, weights of inguinal and gonadal fat depots were ~50 % less in *A-Mttp*^*−/−*^ mice (Fig. 3d). Female and male *Mttp*^*fl/fl*^ control mice gained significant weight when fed HFD (Fig. 3e-f), but *A-Mttp*^*−/−*^ mice did not despite similar amounts of food consumption and body temperature (Fig. 3g-h). These studies indicated that *A-Mttp*^*−/−*^ mice are resistant to diet-induced obesity and accumulate less fat in their adipose tissue.

**Fig. 4:**
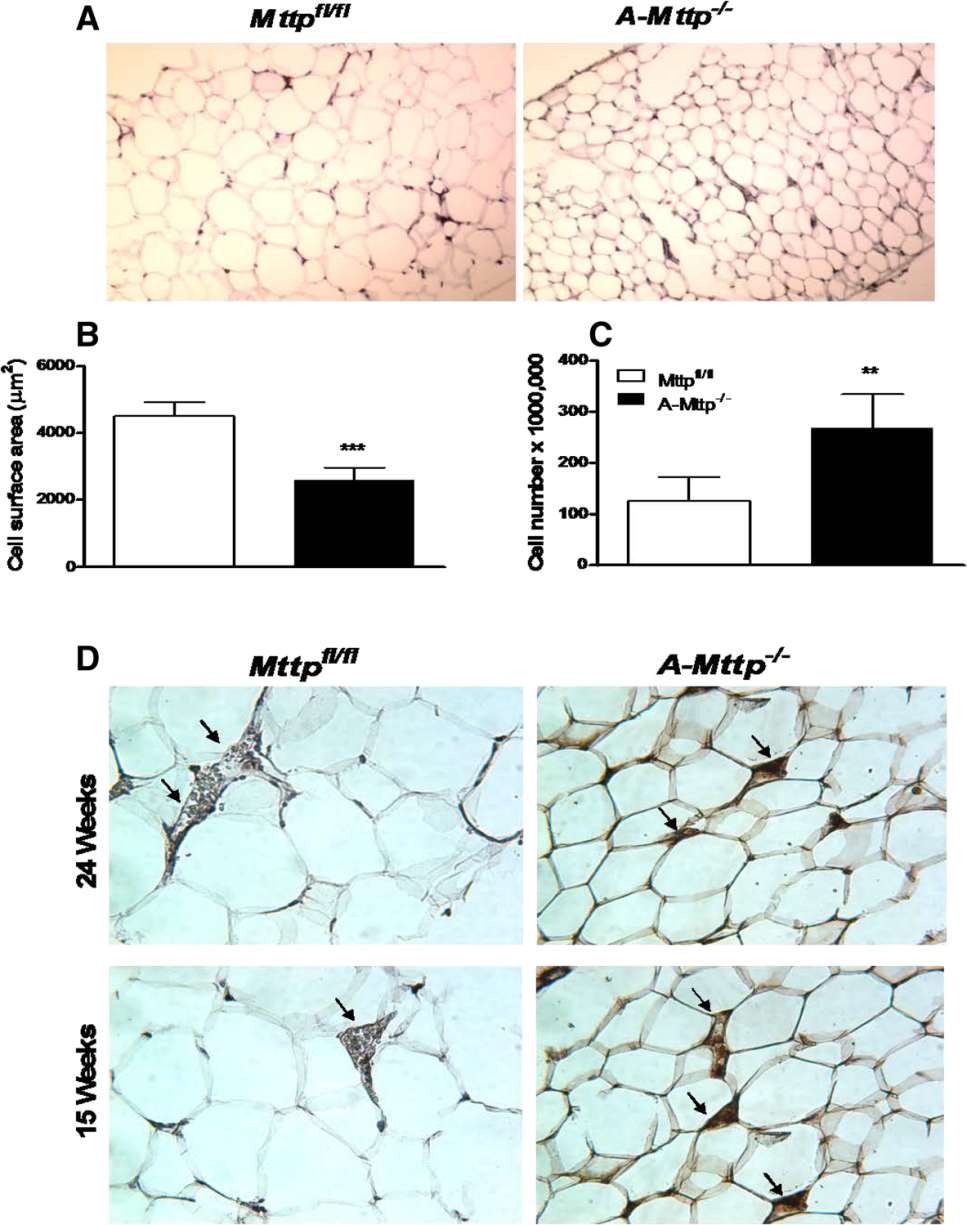
*A-Mttp* ^*−/−*^ mice have smaller size adipocytes and less macrophages infiltration in the adipose tissue. Control *Mttp* ^*fl/fl*^ and *A-Mttp* ^*−/−*^ mice were fed 60 % high fat diet for 15 and 24 weeks. At the end of experiment, adipose tissue was collected for H & E staining (**a**), adipocyte size determination (**b**), and cell number quantification (**c**). Adipose tissue was isolated after collagenase treatment and counted under microscope. Immunohistochemical detection of cells expressing the macrophage-specific antigen F4/80 (arrows) was performed on paraffin section of adipose tissue in control *Mttp* ^*fl/fl*^ and *A-Mttp* ^*−/−*^ mice (**d**)

We then characterized the adipose tissue in these mice. *A-Mttp*^*−/−*^ mice had smaller size adipocytes (Fig. 4a). Adipocytes isolated from inguinal and gonadal fat of *A-Mttp*^*−/−*^ mice had an average size of 2585 μm^2^ as compared to 4500 μm^2^ of the control mice (Fig. 4b). Further, the number of adipocytes obtained from *A-Mttp*^*−/−*^ inguinal fat was greater than the number obtained from WT mice (Fig. 4c). Thus, MTP deficiency increases the number of smaller adipocytes.

Increased macrophage infiltration in the adipose tissue is a common feature of obesity. Hence, we assessed whether adipose tissue macrophage infiltration was altered in *A-Mttp*^*−/−*^ mice. WT adipose tissue showed significant staining for macrophage specific-antigen F4/80 after 15 weeks and 24 weeks of HFD feeding (Fig. 4d). In contrast, the adipose tissue from the *A-Mttp*^*−/−*^ mice stained less for the macrophage antigen. These studies indicated that the adipose tissue of the *A-Mttp*^*−/−*^ mice had less macrophage infiltration.

In short, these studies suggested that *aP2-Cre* mediated MTP ablation results in a leaner phenotype secondary to the presence of greater number of smaller size adipocytes with less number of macrophages in HFD fed mice.

### Ablation of MTP reduces PPARγ expression and its downstream target genes

**Fig. 5:**
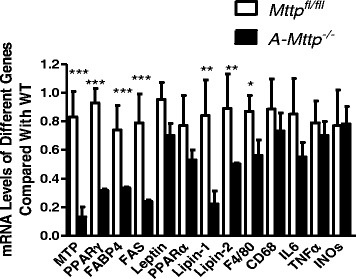
*aP2-Cre* mediated MTP ablation reduces expression of genes involved in adipogenesis. Gene expression in the adipose tissue of *Mttp* ^*fl/fl*^ and *A-Mttp* ^*−/−*^ mice was quantified by qPCR and levels were normalized to 18S RNA. Mice were fed HFD for 24 weeks. After overnight fasting, animals were sacrificed, adipose tissues collected, and isolated inguinal fat adipocytes were used for total RNA extraction using Trizol reagent. cDNA was synthesized and levels of RNA were determined by qPCR. Data are represented as mean ± SD (*N* = 4). *, *p* < 0.05; **, *p* < 0.01 and ***, *p* < 0.001

To understand reasons for the lean phenotype, we measured mRNA levels of different genes that play a role in adipocyte differentiation (Fig. 5). As anticipated, MTP mRNA levels were significantly lower in the adipose tissue of *A-Mttp*^*−/−*^ mice. We also found significant reductions in PPARγ, FABP4, FAS and lipins; other mRNA levels did not differ in two groups. This suggests that MTP deficiency might reduce PPARγ expression resulting in reduced differentiation and lower fat storage in the adipose tissue.

We then measured F4/80, a macrophage marker, and found to be significantly less in *A-Mttp*^*−/−*^ mice indicating less macrophage infiltration (Fig. 5). These studies indicate that *A-Mttp*^*−/−*^ mice were deficient in adipocyte differentiation and macrophage markers.

### A-Mttp^−/−^ mice show normal blood glucose and insulin levels

**Fig. 6:**
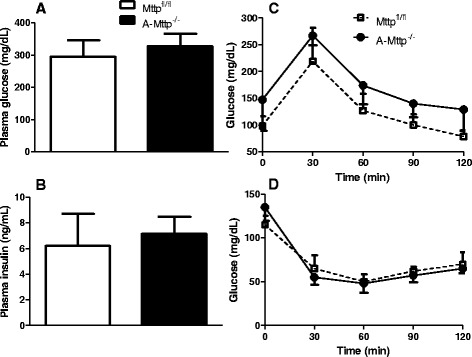
*A-Mttp* ^*−/−*^ mice show normal glucose metabolism. Plasma glucose (**a**) and insulin (**b**) levels were measured in overnight fasted HFD fed animals. Glucose (**c**) and Insulin (**d**) tolerance tests were performed in *Mttp* ^*fl/fl*^ and *A-Mttp* ^*−/−*^ mice fed a HFD for 15 weeks. For glucose tolerance test, mice were fasted overnight (16 h) before glucose challenge (2 mg/kg body weight, i.p.). For insulin sensitivity test, mice were fasted for 4 h before ip insulin injection. Tail blood was collected before injection and after each 30 min up to 2 h. Data are represented as mean ± SD (*N* = 4). *, *p* < 0.05

Next, we concentrated on glucose metabolism in *A-Mttp*^*−/−*^ mice. Fasting glucose and insulin levels did not differ significantly between WT and *A-Mttp*^*−/−*^ mice (Fig. 6a-b). In addition, there were no significant differences in the plasma glucose levels between WT and *A-Mttp*^*−/−*^ mice following glucose and insulin tolerance tests (Fig. 6c-d). Thus, *A-Mttp*^*−/−*^ mice showed no abnormality in glucose metabolism.

### A-Mttp^−/−^ mice have higher levels of plasma triglyceride and normal levels of cholesterol

**Fig. 7:**
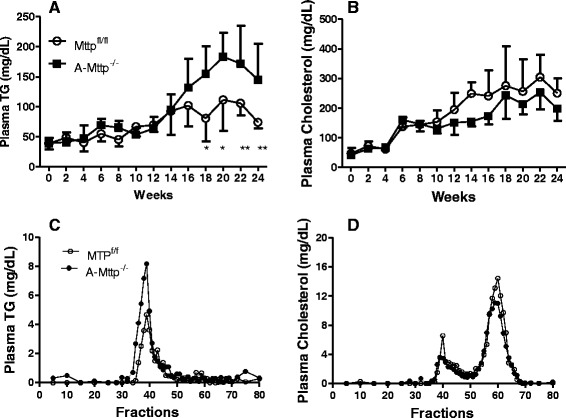
Increased plasma triglycerides in A*-Mttp* ^*−/−*^ mice. Control *Mttp* ^*fl/fl*^ and *A-Mttp* ^*−/−*^ mice were fed 60 % HFD for 24 weeks. Blood samples were collected every 2 weeks and assessed for plasma triglycerides (**a**) and cholesterol (**b**) levels. Data are represented as mean (*N* = 5). Statistical differences between the two groups were obtained by one-way ANOVA analysis. ***, *p* < 0.05; **, *p* < 0.01 and ***, *p* < 0.001. Fasting plasma pool was obtained from five mice in each group and applied to FPLC column for lipoprotein separation. Triglycerides (**c**) and cholesterol (**d**) were measured in each fraction

HFD significantly increased fasting plasma triglyceride by week 18 and this increase continued until week 24 in *A-Mttp*^*−/−*^ mice (Fig. 7a). Fasting plasma cholesterol (Fig. 7b) and free fatty acids levels were similar in these two groups (not shown). Gel filtration analysis of fasting plasma pools from the two groups revealed an increase of triglyceride in the VLDL/LDL fraction of *A-Mttp*^*−/−*^ mice (Fig. 7c) with no significant effect on cholesterol in both VLDL/LDL and HDL fractions (Fig. 7d). These studies suggest that *aP2-Cre* mediated ablation of MTP increases plasma triglyceride-rich lipoproteins.

### A-Mttp^−/−^ mice have increased adipose lipogenesis with no effect on β-oxidation

**Fig. 8:**
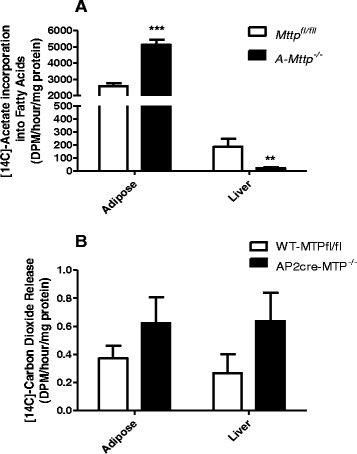
*A-Mttp* ^*−/−*^ mice show enhanced adipose lipogenesis and reduced hepatic lipogenesis without any effect on β-oxidation. *De novo* lipogenesis (**a**) and Fatty acid β-oxidation (**b**) were performed in Control *Mttp* ^*fl/fl*^ and *A-Mttp* ^*−/−*^ mice after a 15 weeks HFD treatment. Adipose tissue and liver were collected from overnight fasted mice (~15 h) and rate of lipogenesis and fatty oxidation were measured. *, *p* < 0.05; **, *p* < 0.01 and ***, *p* < 0.001; and data are mean ± SD (*N* = 4)

We next studied the rate of *de novo* lipogenesis (DNL) and fatty acid oxidation by measuring incorporation of radiolabeled acetate and release of CO_2_, respectively. Adipose tissues from *A-Mttp*^*−/−*^ mice incorporated significantly higher amounts of acetate in newly synthesized fatty acids as compared to control *Mttp*^*fl/fl*^ mice (Fig. 8a). In contrast, hepatic DNL was significantly decreased in *A-Mttp*^*−/−*^ mice as compared to control mice. Fatty acid oxidation in adipose tissue and the liver was unchanged in *A-Mttp*^*−/−*^ mice as compared to control mice (Fig. 8b). These studies suggest that *aP2-Cre* mediated adipose MTP deficiency induces DNL in the adipose tissue but not in the liver.

### A-Mttp^−/−^ mice have lower intestinal triglyceride secretion after an acute dietary fat challenge

**Fig. 9:**
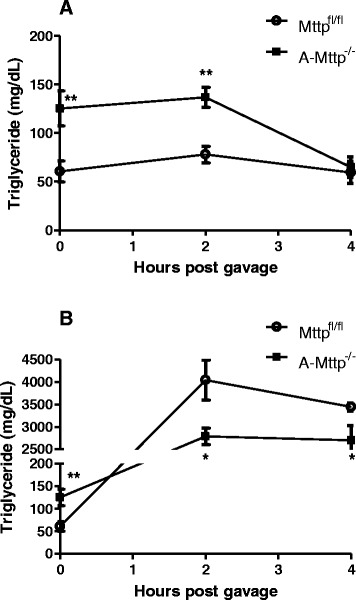
A*-Mttp* ^*−/−*^ mice absorb fewer lipids after a fat tolerance test. Control *Mttp* ^*fl/fl*^ and *A-Mttp* ^*−/−*^ mice were fed 60 % HFD for 15 weeks. (**a**) Postprandial triglyceridemic response was measured in control *Mttp* ^*fl/fl*^ and *A-Mttp* ^*−/−*^ mice. Plasma triglyceride concentration was measured at baseline (4 h fasting) and at 2 h and 4 h following oral challenge of 200 μl olive oil. Data are represented as mean ± SD (*N* = 3). (**b**) After 4 h fasting, mice were injected with P407 in the intraperitoneal cavity to block lipase activity in the circulation. After 30 min, mice were administered 200 μl olive oil (oral gavage) and plasma triglycerides were measured before and at 2 h and 4 h post oil bolus. Data are represented as mean ± SD (*N* = 4). *, *p* < 0.05 and **, *p* < 0.01

To determine whether postprandrial triglyceridemic response was altered in lean *A-Mttp*^*−/−*^ mice compared to *Mttp*^*fl/fl*^ control mice, we measured plasma triglycerides concentration before and after 2 h and 4 h post olive oil bolus. At baseline, levels of plasma triglycerides were higher in lean *A-Mttp*^*−/−*^ mice compared to *Mttp*^*fl/fl*^ control mice as expected, and remained high at 2 h then dropped at 4 h post olive oil gavage (Fig. 9a). Percent increase in plasma triglyceride in the two groups were similar at 2 h (*A-Mttp*^*−/−*^ : 109.20 % ± 8.33 % vs. *Mttp*^*fl/fl*^: 128.75 % ± 14.25 %; *p* = 0.1109) but decreased significantly after 4 h in *A-Mttp*^*−/−*^ mice (*A-Mttp*^*−/−*^ : 51.76 % ± 8.45 % vs. *Mttp*^*fl/fl*^: 98.16 % ± 18.71 %; *p* = 0.0173). To determine whether these changes are secondary to lipoprotein production, we repeated the same experiment in mice injected with P407 to inhibit lipoprotein lipase. As expected, basal plasma triglycerides were higher in *A-Mttp*^*−/−*^ mice compared to *Mttp*^*fl/fl*^ control mice at baseline. *A-Mttp*^*−/−*^ mice had significantly lower amounts of triglycerides at 2 h and 4 h as compared to *Mttp*^*fl/fl*^ control mice (Fig. 9b). The percent increase in plasma circulating triglycerides was significantly lower at 2 h (*A-Mttp*^*−/−*^ : 2084.79 % ± 139.75 % vs. *Mttp*^*fl/fl*^: 6326.82 % ± 705.39 %; *p* < 0.0001) and 4 h (*A-Mttp*^*−/−*^ : 2019.56 % ± 284.54 % vs. *Mttp*^*fl/fl*^: 5388.21 % ± 940.34 %; *p* = 0.0004) in *A-Mttp*^*−/−*^ mice. We calculated the areas under the curves (AUC) and found that in the absence of P407, *Mttp*^*fl/fl*^ control mice had lower AUC value (AUC_0–4_ = 276.09 ± 39.46) as compared to *A-Mttp*^*−/−*^ mice (AUC_0–4_ = 464.13 ± 49.50). In contrast, after blocking the lipase activity in the circulation, the AUC value of *Mttp*^*fl/fl*^ control mice was elevated (AUC_0–4_ = 11587.17 ± 1001.84) compared to *A-Mttp*^*−/−*^ mice (AUC_0–4_ = 8397.79 ± 723.81). These studies indicate that *A-Mttp*^*−/−*^ mice absorb lower amounts of triglyceride suggesting that enhanced plasma triglycerides in lean *A-Mttp*^*−/−*^ mice under HFD is not due to alterations in triglycerides secretion by intestine.

### A-Mttp^−/−^ mice are protected from HFD induced hepatic steatosis

**Fig. 10:**
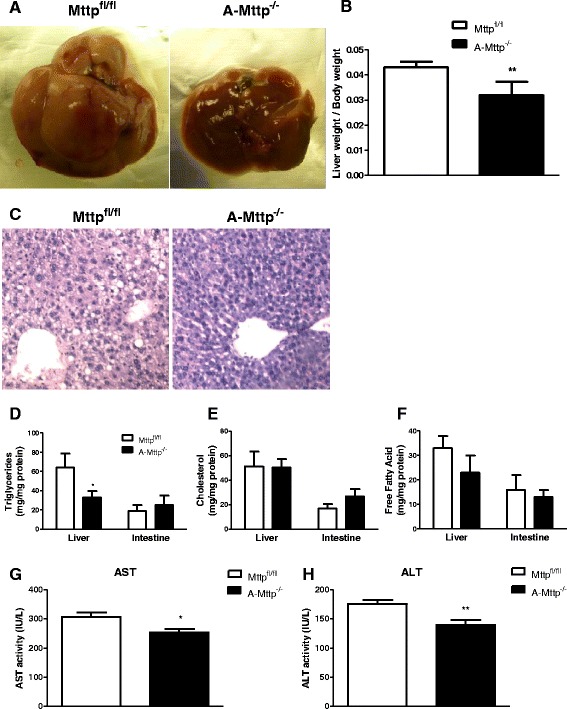
*A-Mttp* ^*−/−*^ mice are protected from HFD induced fatty liver. Control *Mttp* ^*fl/fl*^ and *A-Mttp* ^*−/−*^ mice were fed 60 % HFD for 24 weeks. At the end of an experiment, overnight fasted animals were scarified and livers were collected (**a**), weighed (**b**) and fixed in paraffin and stored at −80 °C until use for H & E staining (**c**). Liver and intestine were homogenized and analyzed for triglyceride (**d**), cholesterol (**e**), and free fatty acid (**f**). Transaminase activities were measured on fasting plasma from five mice (**g & h**). ***, *p* < 0.05; **, *p* < 0.01 and ***, *p* < 0.001; and data are mean ± SD (*N* = 7)

As expected, livers of WT mice fed a HFD were pale indicating fat accumulation (Fig. 10a). In contrast, livers of *A-Mttp*^*−/−*^ mice appeared normal. Consistent with this observation, livers from the WT mice weighed more than those from the *A-Mttp*^*−/−*^ mice (Fig. 10b). Hematoxylin and eosin staining showed more vacuoles in the livers of WT mice (Fig. 10c). Further, lipid analysis revealed that livers obtained from WT mice had higher amounts of triglyceride compared with *A-Mttp*^*−/−*^ mice (Fig. 10d). In contrast to the liver, accretions of triglyceride in the intestine were not different in these two groups. Hepatic cholesterol and free fatty acid contents were not different in these two groups of mice (Fig. 10e-f). Plasma transaminases are used to ascertain liver damage and function (34, 42). Interestingly, protection from hepatic steatosis in the *A-Mttp*^*−/−*^ mice was associated with significant decrease of plasma AST and ALT (Fig. 10g-h). These studies showed that *A-Mttp*^*−/−*^ mice exhibit less hepatosteatosis compared to WT mice on a HFD.

## Discussion

In this study, we asked whether MTP plays a role in adipocyte biology. Our studies showed that MTP is induced early during differentiation of 3T3-L1 cells before the induction of PPARγ, a critical transcription factor for the differentiation of adipocytes. Further, we show for the first time that *A-Mttp*^*−/−*^ mice are lean and resistant to diet-induced obesity when fed a high fat diet. The adipose tissue in these mice contained more number of smaller size adipocytes and had less macrophage infiltration. These studies suggest that MTP plays a role in determining size, number, and triglyceride content of adipocyte most likely by assisting in greater accretion of triglyceride and enhancing the size of adipocyte in high fat fed mice.

MTP expression changes during differentiation of 3T3-L1 preadipocytes into adipocytes. Undifferentiated 3T3-L1 preadipocytes had lipid transfer activity suggesting for the presence of pre-existing pools of MTP. *Mttp* gene was induced early during differentiation that preceded transcriptional regulation of key markers of differentiation such as PPARγ consistent with other studies [21]. Mechanistic studies by other investigators have suggested that the induction of MTP in early differentiation might be due to the binding of C/EBPβ and C/EBPδ transcription factors to a specific region in the exon 1 of the *MTTP* gene [21]. Thus, MTP is an early response gene involved in adipocyte differentiation.

Our study shows that early expression of MTP during differentiation is critical for optimal lipid storage. Molecular approaches using siRNA-mediated knockdown of MTP and overexpression of MTP demonstrated that changes in MTP affect intracellular lipid storage and droplet formation (Fig. 2). Overexpression of MTP increases the accretion of triglyceride in differentiated 3T3-L1 cells, whereas knockdown reduces their accumulation. These data are consistent with those of Rakhshandehroo et al. [21] who reported that MTP depletion (stable shRNA knockdown of MTP) does not influence the differentiation of 3T3-L1 preadipocytes, as assessed by FABP4 expression. These data suggest that MTP plays a role in promoting adipogenesis and determines the amounts of lipids accreted by differentiated adipocytes. In contrast to these studies, MTPi did not interfere with triglyceride accumulation. Thus MTP protein, not its lipid transfer activity, might play a role during adipocyte differentiation by assisting in the fusion of small droplets into larger droplets.

Surprisingly, treatment of cells with MTPi did not affect adipocyte differentiation and adipogenesis (Fig. 1). This is consistent with a recent finding demonstrating that the lipid transfer activity of MTP is neither critical for the mobilization of fatty acids from adipocytes nor for cell differentiation as assessed by the number of intracellular lipid droplets [44]. Our findings are also in agreement with another study dealing with the CD1d-mediated lipid self-antigen presentation in adipocytes showing that treatment of adipocytes with BMS-212122 at high dose (13 μM) for 24 h did not influence the differentiation potential of the preadipocytes [21]. Taken together, our results and observations from others suggest that MTP protein, but not its lipid transfer activity, may play a pivotal role in adipocyte lipid droplet formation and maturation. It is possible that MTP protein might be involved in the fusion of smaller lipid droplets to from larger droplets during adipocyte differentiation.

Understanding the precise function of MTP during adipocyte differentiation is critical in delineating its role in larger lipid droplet formation. MTP might participate in lipid accumulation via other mechanisms. Analysis of the expression pattern of relevant genes controlling adipocyte differentiation and adipogenesis identified that the induction of PPARγ was reduced in *A-Mttp*^*−/−*^ mice and the maximum expression of MTP occurs prior to the maximum expression of PPARγ in 3T3-L1 cells. PPARγ is known to activate nearly all of the genes required for the formation of larger droplets, including FABP4 which is required for the transport of free fatty acids, and perilipin which is on the surface of mature lipid droplets. Thus, it is possible that reduced expression of PPARγ might be one mechanism contributing to reduced fat accumulation in MTP deficient adipocytes.

Another possibility is that MTP might interact with lipid droplet associated proteins, such as perilipin, and assist in droplet formation and/or stabilization of larger droplets. Immunohistochemical studies have shown that MTP surrounds small lipid droplets in 3T3-L1 adipocytes as well as in white and brown fat in mice [14, 15]. MTP has been found to physically interact with apoB in lipoprotein producing cells [10, 18]. Therefore, it is possible that MTP may interact with another protein(s) in adipocytes and act as a chaperone. For example, MTP could interact with one or more structural proteins on the surface of lipid droplets such as perilipin, adipophilin, TIP47, OXPAT/MLDP, and S3-12 [45–47], and assist in droplet fusion process by providing close access to triglycerides for smaller droplets, promoting lipid droplet growth and expansion. In fact, Love et al. demonstrated that MTP and perilipin-2 were associated with the same isolated lipid droplets from adipocytes, but they concluded that these proteins do not interact with each other [44]. More studies are needed to explore possible physical interactions between MTP and lipid droplet-coating proteins.

Our finding of lean phenotype associated with reduced fat pad weight and lack of an increase of adipocyte size in HFD fed *A-Mttp*^*−/−*^ mice hints at a role of MTP in modulating adipocytes differentiation. Interestingly, reduction of fat mass in *A-Mttp*^*−/−*^ mice was accompanied with increased number of smaller adipocytes (Figs. 3 and 4). Thus, it is likely that basal cellular levels of triglycerides are unaffected by MTP. However, increased accumulation of fat during diet-induced obesity may require MTP. This is supported by the observation that *A-Mttp*^*−/−*^ mice on chow do not show any difference in body weight compared to controls.

Metabolic defects that alter adipose tissue fat accumulation are frequently associated with changes in glucose homeostasis. Unexpectedly, plasma glucose and insulin levels in the fasted HFD-fed animals did not differ between the *A-Mttp*^*−/−*^ and WT mice. Further, despite marked reduction in body weight and adiposity, *A-Mttp*^*−/−*^ mice did not exhibit any changes in whole-body glucose homeostasis and insulin sensitivity (Fig. 6). The reasons for no effect on glucose metabolism are not clear.

Using 1-^14^C acetate as substrate, we observed that the adipose tissue and liver accounted for about 93 % and 7 % of DNL in WT mice. This in agreement with previous studies suggesting that adipose tissue DNL is a major site of lipogenesis in rodents [48–50]. In *A-Mttp*^*−/−*^ mice, we found that DNL was significantly higher in the adipose tissue and was lower in the liver (Fig. 8). This is in good agreement with study showing significant elevation of adipose DNL in ob/ob mice in comparison with control mice [51]. Numerous studies indicated that enhanced adipose DNL, in contrast to the liver DNL, may be beneficial for whole-body metabolism. For example, liver-specific deletion of SCAP, a protein required for the cleavage of SREBP1c to its active form, has been shown to reduce hepatic lipogenesis and enhance adipose DNL by four-fold resulting in improved glucose homeostasis [52]. In another study, ablation of adipocyte/macrophage lipid chaperones *aP2* (FABP4) and *mal1* (FABP5) increased adipose DNL which protected mice from diet-induced obesity, fatty liver disease and insulin resistance [53]. The ob/ob mice that were rendered LPL deficient in adipose tissue also demonstrated increased DNL and diminished weight and fat mass [54]. Similar to these models, our finding suggest that increased DNL in adipocytes is associated with lean phenotype. The fact that DNL is upregulated while β-oxidation is decreased indicates for a possible higher rate of intracellular lipolysis and secretion of fatty acid from adipose tissue of lean *A-Mttp*^*−/−*^ mice. More experiments are needed to illustrate potential impact of relevant lipases on the release of fatty acids and glycerol by adipose tissues.

Accumulation of adipose tissue macrophages has been well described in obese conditions in mice and humans [37, 55–57]. This process of macrophage infiltration is associated with adipocyte hypertrophy and metabolic dysfunction. In addition to the smaller size of adipocytes, we observed lesser macrophage infiltration in the adipose tissue of *A-Mttp*^*−/−*^ mice (Fig. 4). Thus, MTP deficiency in *A-Mttp*^*−/−*^ mice protects adipose tissue from HFD-induced macrophage infiltration.

Our studies also provide evidence that MTP deficiency in *A-Mttp*^*−/−*^ mice modulates the expression of genes involved in adipocyte differentiation and lipid formation and storage. The expression of PPARγ and its downstream targets FAS, lipins and FABP4 were significantly reduced (55 %-75 %) in the adipose tissue of *A-Mttp*^*−/−*^ mice. PPARγ is essential for adipocyte viability and regulates a number of genes involved in lipid uptake and storage [35, 58, 59]. Down regulation of PPARγ could provide protection from HFD induced inflammation and obesity [60–65]. It remains unclear how ablation of MTP in *A-Mttp*^*−/−*^ mice impacts transcription of PPARγ and protects mice from HFD induced inflammation and obesity.

Although *A-Mttp*^*−/−*^ mice consumed similar amounts of food, they did not accumulate fat in the adipose tissue and the liver compared with WT mice. Further, we did not find differences in rectal temperatures of these mice (Fig. 3). Thus, the physiologic reason for the leaner phenotype in these mice is not clear. It is possible that the *A-Mttp*^*−/−*^ mice oxidize fat more efficiently and have higher metabolic rates than the WT mice. Future studies may identify the physiologic reasons for the leaner phenotype in *A-Mttp*^*−/−*^ mice when challenged with a high fat diet.

*A-Mttp*^*−/−*^ mice have higher plasma triglyceride (Fig. 7). These mice showed modest increase (~28 %) in hepatic MTP activity but mRNA levels did not change (Fig. 3). Thus, changes in hepatic MTP do not explain increases in plasma triglyceride in *A-Mttp*^*−/−*^ mice. Further, intestinal lipoprotein assembly and secretion was reduced, not increased. Therefore, increased lipoprotein production is unlikely to be responsible for higher plasma triglyceride in these mice. In addition, postprandial secretion of triglycerides in response to acute fat challenge was significantly decreased in *A-Mttp*^*−/−*^ mice after a 4 h oral fat tolerance test. These mice absorbed lesser amounts of fat than control. Further, we observed reduced lipid absorption in lipase inhibited *A-Mttp*^*−/−*^ mice (Fig. 9). It is plausible that triglyceride elevations might be secondary to reduced amounts of lipoprotein lipase. This is based on several studies that show that adipocyte lipoprotein lipase is reduced in high fat fed animals. In fact, in recent study [66], mice lacking prostaglandin E receptor subtype 4 (EP4) manifested disrupted lipid metabolism associated with a 69 % reduction in weight gain and fat mass following HFD feeding. Plasma triglycerides in these mice were elevated by 245 %. Authors found that these mice had reduced lipoprotein lipase activity, the key enzyme responsible for trafficking of plasma triglycerides into peripheral tissues.

The data obtained with 3T3-L1 cells and high fat fed *A-Mttp*^*−/−*^ mice appear to provide different results. If we assume that 3T3 cell culture studies are comparable to chow fed mice, then results in cells and mice are the same. Both cells and mice do not show any significant difference in fat accumulation during MTP deficiency. We only saw the effect of MTP deficiency when mice were fed a high fat diet. We did not do similar fat challenge studies in cells. Thus, adipose MTP might be more critical when cells are exposed to high lipids.

We are aware that adipocytes express two MTP isoforms, A and B. Dr. Swift’s laboratory was the first to establish the presence of MTP in adipose tissue as well as in the pre-adipocyte cell line, 3T3-L1 [14]. The group has previously generated anti-MTP antibodies from a 19-amino acid peptide representing residues 843 through 861 in the MTP protein. In a recent report [44], they were able to monitor mRNA and protein levels of both the MTP isoforms in adipocytes. They showed that adipocytes predominantly (>95 %) express MTP B isoform and demonstrated that MTP protein expression increased linearly over the 8-day period of differentiation, but mRNA levels for MTP-A and MTP-B did not change significantly. In another study, MTP-B mRNA levels increased significantly within the first two days of differentiation and decreased slightly afterwards [21]. This later report is in good agreement with our observation for the decline of MTP expression at late stages of differentiation. Therefore, it is likely that MTP-B is the major isoform in adipocyte. Further, it should be pointed that no functional differences between these two isoforms have been reported. Therefore, expressions of these two different isoforms as determined by mRNA or protein levels do not appear to have any functional consequences.

In this study, we used *aP2-Cre* to ablate the *Mttp* gene. In these mice, MTP expression was greatly diminished in isolated adipocytes (>80 %) and decreased by ~ 50 % in adipose tissue which also includes non-adipocyte cell types. We are aware that *aP2-Cre* is expressed in adipocytes and macrophage. Therefore, the observed effects might be due to the deletion of MTP in both the adipose tissue and macrophages. In our study, we found significant reductions in the adipose tissue MTP but not in the spleen, a tissue rich in macrophages. Thus, it is likely that the observed effects are due to MTP deficiency in the adipose tissues. It is unlikely that reduction in T-cell mediated immune response due to possible deletion of MTP in macrophages could account for the lean phenotype and metabolic changes observed in *A-Mttp*^*−/−*^ mice for the following reasons: (1) iNKT cell population and CD1d expression is reduced in the adipose tissue of obese mice and humans compared to those of lean subjects [67]; (2) iNKT cell-specific Jα18 knockout mice are obese and exhibit increased adipose tissue inflammation at the early stage of obesity [68]; (3) CD1d-deficient mice fed a high-fat diet show insulin resistance and hepatic steatosis [69–71]. Future studies may dissect the role of MTP in the adipose tissue and macrophages using cell-specific deletion of the gene.

## Conclusion

In short, our study provides the first evidence of the role played by MTP in adipocyte differentiation and fat storage in mice. Ablation of the *Mttp* gene using *aP2-Cre* has no effect on glucose homeostasis but it prevents HFD induced hepatosteatosis. We present evidence that MTP protein plays a role in the accumulation of larger lipid droplets in the adipose tissue. We speculate that MTP may (1) activate expression of PPARγ and amplify expression of genes critical for adipocyte maturation and lipid storage, (2) interact with lipid droplet associated proteins, and/or (3) assist in the fusion of smaller lipid droplets. Future investigation of how MTP ablation could repress PPARγ transcriptional activity should provide additional insights into the role of MTP in adipocyte maturation and physiology. It is possible that down regulation of adipose MTP might avoid complications associated with obesity.

## Abbreviations

ALT: alanine aminotransferase
AP2: adipocyte protein 2
ApoB: apolipoprotein B
AST: aspartate aminotransferase
CD1: cluster of differentiation 1
DNL: de novo lipogenesis
ER: endoplasmic reticulum
FABP4: fatty acid binding protein 4
FAS: fatty acid synthase
FBS: fetal bovine serum
GTT: glucose tolerance test
HFD: high-fat diet
IBMX: 3-isobutyl-1-methylxanthine
ITT: insulin tolerance test
MTP: Microsomal triglyceride transfer protein
NKT: natural killer T cells
PPARγ: peroxisome proliferator-activated receptor gamma
TG: triglycerides
